# The association of lipids with amyloid fibrils

**DOI:** 10.1016/j.jbc.2022.102108

**Published:** 2022-06-08

**Authors:** John M. Sanderson

**Affiliations:** Department of Chemistry, Durham University, Durham, United Kingdom

**Keywords:** amyloid, lipid, fibrillogenesis, synuclein, hIAPP, tau, prion, Aβ, Aβ(1–42), fragment 1 to 42 of the amyloid-β peptide, AD, Alzheimer’s disease, GM1, monosialotetrahexosylganglioside, GUV, giant unilamellar vesicle, hIAPP, human islet amyloid polypeptide, LB, Lewy body, MS, mass spectrometry, PC, phosphatidylcholine, PI, phosphatidylinositol, P:L, protein to lipid ratio, PM, plasma membrane, POPG, 1-palmitoyl-2-oleoyl-*sn*-glycero-3-phosphoglycerol, PrP, prion protein, PrPSc, scrapie prion protein, PTM, post-translational modification, SM, sphingomyelin, ssNMR, solid-state NMR, α-Syn, α-synuclein

## Abstract

Amyloid formation continues to be a widely studied area because of its association with numerous diseases, such as Alzheimer’s and Parkinson’s diseases. Despite a large body of work on protein aggregation and fibril formation, there are still significant gaps in our understanding of the factors that differentiate toxic amyloid formation *in vivo* from alternative misfolding pathways. In addition to proteins, amyloid fibrils are often associated in their cellular context with several types of molecule, including carbohydrates, polyanions, and lipids. This review focuses in particular on evidence for the presence of lipids in amyloid fibrils and the routes by which those lipids may become incorporated. Chemical analyses of fibril composition, combined with studies to probe the lipid distribution around fibrils, provide evidence that in some cases, lipids have a strong association with fibrils. In addition, amyloid fibrils formed in the presence of lipids have distinct morphologies and material properties. It is argued that lipids are an integral part of many amyloid deposits *in vivo*, where their presence has the potential to influence the nucleation, morphology, and mechanical properties of fibrils. The role of lipids in these structures is therefore worthy of further study.

There is a vast body of literature describing the self-aggregation and assembly of proteins into oligomers and insoluble fibrils rich in β-structure. This aggregation is associated with numerous conditions, including, amongst others, Parkinson’s disease, Alzheimer’s disease (AD), and Huntingdon’s disease, and type II diabetes. Studies cover a range of fibril behavior, including their growth *in vitro* from purified proteins, the composition and structural features of deposits found *in vivo*, and *ex vivo* study of the molecular characteristics of fibrils isolated from natural sources. However, whilst there has been a large focus on the protein elements of these aggregates, in living tissues, these proteins are usually deposited alongside other components, including lipids, carbohydrates (1), metal ions, and even whole organelles in some cases, such as dementia with Lewy bodies (LBs) (2). It is noteworthy that the earliest accounts of amyloid deposits in the 18th and 19th centuries described them as “lardaceous” and “waxy” (3, 4, 5), with the term “amyloid” being adopted much later. In the 1960s, the lipid content of amyloid deposits began to be scrutinized in more detail, often leading to conclusions that lipids were present as a contaminant (6, 7). The focus subsequently shifted to address protein aggregation. In recent years however, in the face of a wealth of data on peptide and protein assembly into fibrils, it has become apparent that membranes have a key role in accelerating assembly *in vivo* and driving assembling intermediates into conformations that are on-pathway for fibril formation (8, 9, 10, 11, 12, 13, 14). But whilst assembly processes have been well characterized under controlled conditions *in vitro*, a number of significant challenges remain in understanding both the processes that occur *in vivo* that lead to otherwise normal functioning peptides and proteins becoming directed down aberrant folding pathways and the parts of this process where cellular damage occurs. A particularly complex challenge is understanding the link between fibrillogenesis and biological outcomes, as it is frequently difficult to relate the *in vitro* behavior to the *in vivo* toxicity. Fibrils formed in the presence of lipids, for example, can exhibit differing morphologies according to the method of preparation (11, 14, 15, 16, 17). For many diseases that involve the formation of amyloid fibrils, the precise physiological circumstances that trigger toxic fibril formation are still poorly characterized, and a number of phenomena lack detailed information at the molecular level, such as how changes in lipid composition are able to influence nucleation kinetics and fibril morphology (18, 19).

There has recently been a shift back toward examining amyloid fibrils in their biological context, including an examination of the presence of other molecular components that may influence their properties or provide evidence that can improve our understanding of their biological function. It is unsurprising that fibrils should be associated with membranes *in vivo*, as this reflects the environment in which they form. Fibers are often associated closely with bound lipids when studied *in vivo* and also sometimes when prepared *in vitro* in the presence of membranes. There are two general types of lipid association with fibrils: loose (easily removed by washing or mechanical disruption) and tight (persisting even after washing). This review is concerned with the nature of lipid association with fibrils and not with the mechanisms underlying fibril nucleation, except where this nucleation is responsible for lipid inclusion in the fibril structure.

## Overview of amyloid assembly

**Figure 1:**
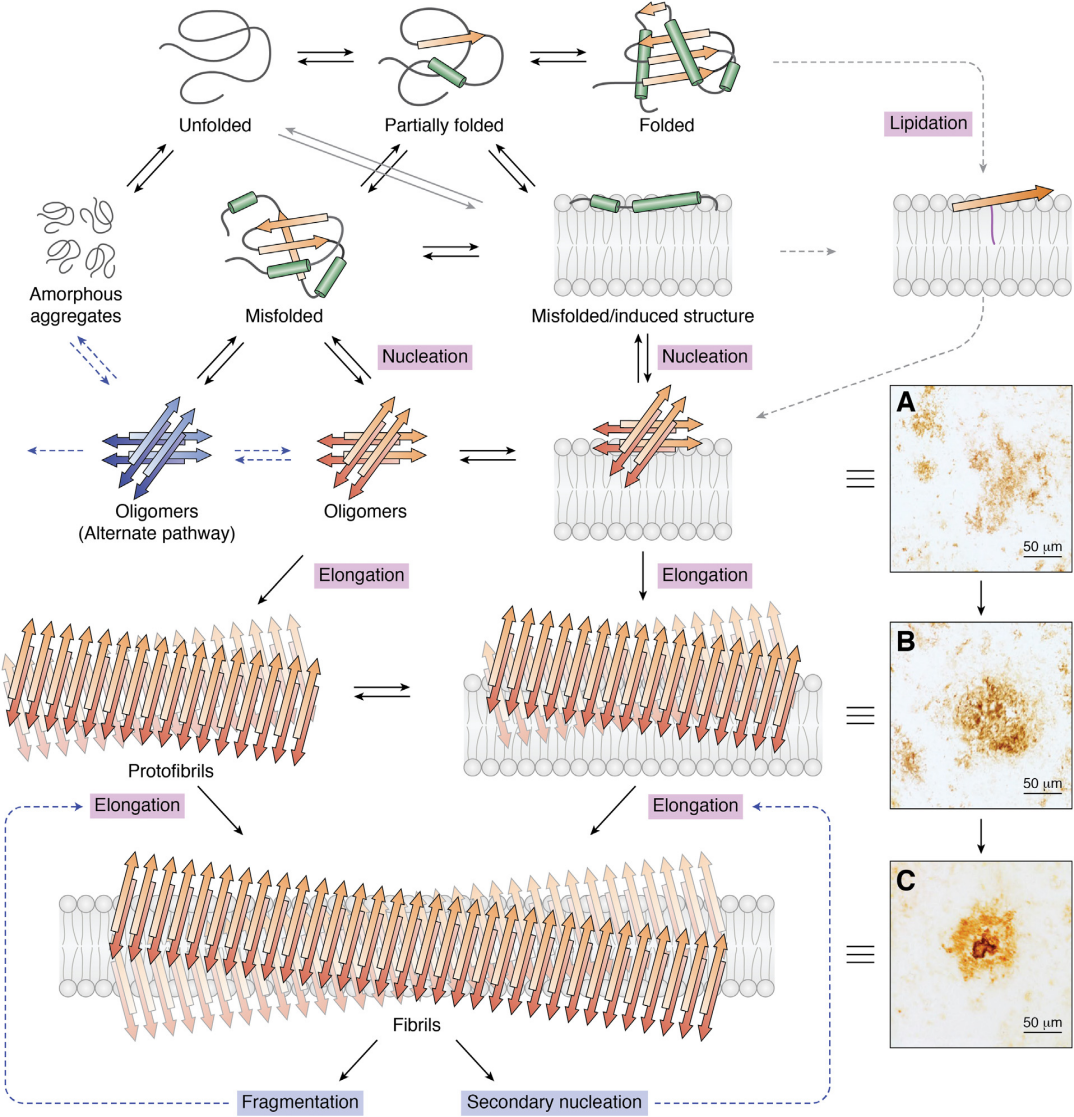
**An overview of the pathways leading to fibril formation.***A*–*C*, adapted from Ref. (53) under a Creative Commons license (CC BY 4.0) and show images of plaques obtained postmortem from the brains of AD sufferers using an immunochemical stain against Aβ. *Dotted arrows* indicate a route hypothesized to lead to nucleation *via* lipidation of a membrane-associated state (35). AD, Alzheimer’s disease.

Several good reviews of fibrillogenesis are available in the literature (10, 16, 20, 21, 22, 23). A generic overview of the processes leading to the formation of fibrils is given in Figure 1. Key to the process is the adoption of misfolded conformations of a protein monomer or a peptide monomer. Brief descriptions of several proteins and peptides that are known to form amyloid fibrils are given in Box 1. In some circumstances, an unfolded protein or a misfolded protein will form amorphous aggregates. Many nonamyloidogenic proteins form amorphous aggregates with lipids (24, 25), and although these structures are not of direct interest to this review, it remains a salient point that it is necessary to demonstrate that a protein–lipid aggregate has a fibrillar structure.

Box 1 **Examples of well-studied proteins and peptides**

**Amyloid Aβ**

Aβ peptides form by processing of the β-amyloid precursor protein by β- and γ-secretases (4, 146). The role of lipids in Aβ fibril formation has been characterized extensively (4, 27, 32, 47, 147), including the influence of membrane composition on fibril morphology (18, 27). Aβ(1–42) is associated with diffuse plaques in the early stages of AD. Deposition begins on the plasma membrane (PM) in close proximity to cell protrusions (processes) and leads to the formation of an amorphous substance. The PM eventually disappears, and the deposits become more fibrillar (55).

**α-Synuclein**

α-Synuclein (α-Syn) is found alongside lipids, carbohydrates, and organelles in LBs (2, 87). The membrane interactions of α-Syn have been well reviewed (9, 148, 149, 150, 151). The peptide is unstructured in solution, becoming helical upon binding of its N-terminal regions to membranes containing anionic lipids (152, 153, 154, 155). Fibril nucleation is strongly promoted in membrane-bound conformations (156, 90), some of which have been hypothesized to arise by peptide lipidation (35). Other PTMs modulate membrane interactions and aggregation (157), and the interplay between lipid homeostasis, mitochondrial function, and fibril formation is complex (158).

**Human islet amyloid polypeptide (amylin)**

The deposition of human islet amyloid polypeptide (hIAPP) fibrils in islet cells is a feature of type II diabetes mellitus (8, 159, 160). As with many amyloid-forming peptides, hIAPP is unstructured in solution, adopting an amphipathic helix when membrane bound (161, 162). Potential binding sites include the mitochondrial envelope (8, 155). Membrane interactions occur at the N terminus of the peptide (159, 163). The presence of membranes accelerates fibril formation and influences fibril morphology (41, 45, 163).

**Tau**

Tau proteins modulate the polymerization dynamics of microtubules in axons (164). In AD, tau becomes hyperphosphorylated and forms intracellular neurofibrillary tangles (165). Binding to membranes containing negatively charged lipids leads to an increase in helicity (166). Some tau variants bind to dimyristoyl-*sn*-glycero-3-phosphoglycerol monolayers and form membrane-bound fibrils (13).

**Scrapie prion protein**

Scrapie prion protein (PrP^Sc^) is the abnormal scrapie form of the cellular prion protein (PrP). PrP is anchored to the cell surface by a glycosylphosphatidylinositol anchor (167), and binding to negatively charged membranes influences its folding (11, 101). PrP^Sc^ is also tightly associated with the membrane (100, 168, 169) but is resistant to detachment by PI-specific phospholipase C (170, 171). In some cases, PrP accumulation around neurons is associated with membrane degradation (172).

**Other proteins**

Other proteins that form aggregates with membrane lipids that are amyloid in nature include apolipoprotein A-II, which forms extracellular fibrils (52); immunoglobulin light chain, amyloid fibrils of which colocalize with lipids and cholesterol *in vivo* (although not *in vitro* or *ex vivo*) (103); and endostatin (173).

Some misfolded conformations are able to nucleate and seed aggregation into extended aggregates rich in β-structure. This nucleation may happen when the monomer is associated with a membrane or in free solution and occurs during the lag phase of fibrillogenesis. It is a fundamental point that there are multiple pathways for fibril formation, many of which lead to fibrils with differing structural and morphological properties (14, 18, 26, 27). The biologically relevant morphology may be different from one produced from a purified protein *in vitro* under a given set of conditions. In many cases, it has been demonstrated that fibril formation is promoted following membrane binding of a protein monomer or a protein oligomer (14, 28, 29, 30, 31, 32). This acceleration is generally attributed to a combination of factors, including the bound protein adopting a more favorable conformation for nucleation, the concentration of the protein into a small volume, and changing the process from one occurring in bulk solution to one that is effectively a 2-dimensional process (28). It is notable that many proteins that are unfolded in solution adopt elements of secondary structure on binding to the membrane, and this is likely to be a key feature in their nucleation (22).

Although folded proteins are not on-pathway for fibril formation, it is nevertheless possible that post-translational changes can lead to modifications that tip the balance in favor of misfolding. The addition of an alkyl chain from 4-hydroxy-2-nonenal, a byproduct of lipid autoxidation, has been found to favor amyloid formation in some cases (33) and prevent fibril formation in others (34), although in the latter case, the peptide was extensively modified. The direct addition of acyl groups (lipidation) has also been proposed to drive some peptides into membrane-anchored conformations competent for fibril formation (35). In a similar manner, when artificially labeled *in vivo* with a glycosylphosphatidylinositol anchor, the fragment 1 to 42 of the amyloid-β peptide (Aβ(1–42)) was found to deposit much more rapidly than the untagged peptide (36). Post-translational modifications (PTMs) such as lipidation may also affect the interfacial interactions between protofilaments as well as packing interactions within the protofilament core.

Following nucleation, at the end of the lag phase, the addition of further monomers to the oligomers leads to the formation of protofibrils, often the first detectable aggregates. Growth at this stage occurs over a period of hours to days and leads to the eventual formation of fibrils. These fibrils may then be subject to further changes, such as fragmentation and secondary seeding, which influence both the assembly kinetics and the final fibril morphology (37, 38). It is now generally recognized that the lower to intermediate molecular weight species, rather than the mature fibrils, are the toxic species that inflict damage to cell membranes (29, 39, 40, 41, 42, 43). Indeed, larger fibrils formed by a number of proteins are nontoxic, and some perform functional roles within the cell (44).

### The role of membranes in fibril formation

Several key features of amyloid nucleation in membranes are influenced by the presence of a membrane. For many peptides, key fibrillation characteristics, such as the duration of the lag phase, the rate of fibril growth and fibril morphology, vary significantly with changes in membrane lipid composition and peptide to lipid ratio. In many cases, nucleation is significantly accelerated in the presence of negatively charged lipids (41, 45, 46). In general, liposome models are able to replicate some of the features of fibrils found *in vivo*, but often the outcomes of experiments with liposomes are dependent on experimental details, such as whether a peptide is added to preformed liposomes or incorporated into the membranes during formation (18, 27, 47, 48). The dependence of the outcome on both the method of preparation and the membrane composition makes comparisons between fibrils formed *in vitro* and those isolated from tissues more challenging.

Oxidized lipids may also play a role in triggering nucleation. In some cases, such as lipids in which an unsaturated *sn*-2 acyl chain has been oxidatively cleaved to form a carboxyl group, these effects on nucleation may be a consequence of the presence of additional negative potential on the membrane surface and changes to the intrinsic curvature of the lipid (49). In some model systems, the presence of oxidized lipids produces a significant change in peptide behavior, leading to the formation of peptide–lipid aggregates with a helical appearance by microscopy as a consequence of significant membrane remodeling during peptide aggregation (49). Amyloid formation may not also be a unique feature of the plasma membrane, as oxidative damage to low-density lipoprotein leads to the formation of Aβ fibrils *in vivo* (50), and exosomes are also involved in the transport of many amyloid-forming proteins around the cell (51). Amyloid formation by proteins associated with high-density lipoprotein and lipid droplets has also been described (52).

## Evidence for the presence of lipids in amyloid fibrils

Multiple studies of amyloid deposits *in vivo*, after isolation from natural sources (*ex vivo*), or formed *in vitro* in the presence of a source of lipids have found evidence for the presence of lipids in fibrillar structures. In the case of AD, brain tissues have been examined by numerous methods, with the most common being confocal imaging combined with vibrational spectroscopy, imaging by mass spectrometry (MS) or related methods, and lipidomics by chromatography combined with MS.

### Studies of Aβ *in vivo*

The early stages of Aβ aggregation are characterized by the formation on the cell surface of diffuse amorphous structures (plaques) containing protein and little or no lipid (53, 54). These plaques typically form in contact with the highly curved cell protrusions of astrocytes or neurons (55, 56), and the protein within appears as randomly dispersed accumulations (Fig. 1*A*) (53). Following the formation of diffuse plaques, subsequent steps lead to a loss of cellular material, visualized as a disintegration of the PM. The assumption is often made that the lipids lost are incorporated into fibrils (55, 57). In one case, the lipids released into the medium were analyzed and found to consist predominantly of cholesterol, phospholipids, and the ganglioside GM1 (monosialotetrahexosylganglioside), with Aβ colocalizing with GM1 after fractionation by density gradient ultracentrifugation (15). The loss of cellular material eventually leads to the formation of compact plaques and eventually classic cored plaques (Fig. 1, *B* and *C*, respectively). The rest of the discussion in this section concerns classic cored plaques containing Aβ.

A detailed review of the lipid content of these plaques, as identified through lipidomics studies, has recently been presented (58). In mouse models, relative to normal tissue, there is an accumulation of fatty acids, cholesterol, sulfatides, and phosphatidylethanolamine lipids in plaques, and a depletion of phosphatidylcholine (PC) lipids. In human tissues, there is an accumulation of numerous lipid species in plaques, including ceramides, gangliosides, lysolipids, and phosphatidylinositol (PI) lipids, alongside a depletion of cerebrosides and sulfatides. For some phospholipid classes, including PC and phosphatidylethanolamine, there is no consistent pattern of enhancement or depletion. These examples demonstrate that for many lipid classes, the data are nuanced, with species-specific changes in lipid levels.

**Figure 2:**
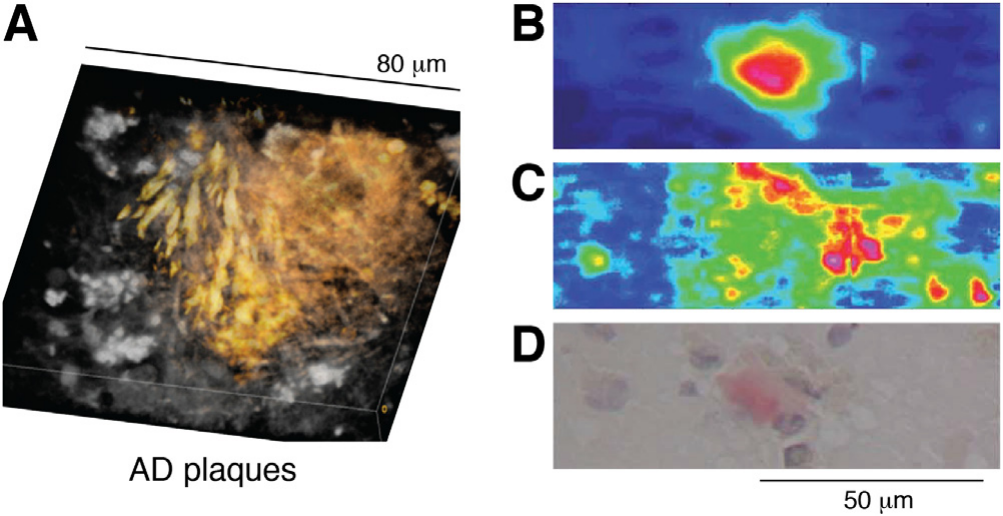
**Imaging of AD plaques *in vivo* to reveal their lipid distributions.***A*, extracted from Ref. (54) under a Creative Commons license (CC BY 4.0) and shows a 3D rendering obtained by coherent anti-Stokes Raman scattering (CARS) showing lipids (in *yellow*) distributed across the plaque in needle-like structures on the *left* and a more diffuse distribution on the *right*. *B*–*D*, adapted from Ref. (60) under a Creative Commons license (CC BY-NC 3.0) and depict different images of the same plaque isolated from a human AD patient. *B* and *C*, obtained by FTIR imaging, with *red* and *blue* corresponding to regions of high and low intensity, respectively. *B*, shows the amide I band, corresponding to β-sheet. *C*, the CH_2_ stretching vibration, arising primarily from lipids. *D*, shows a conventional image, with the plaque core stained using Congo red and the nuclei stained using hematoxylin blue. AD, Alzheimer’s disease.

Fibrillar plaques from patients with AD have been scrutinized using fibril-specific dyes combined with other imaging methods, such as vibrational imaging, to enable both peptide and lipid distributions to be characterized (53, 54, 59, 60). Imaging of the lipid CH and peptide amide vibrations by coherent anti-Stokes Raman scattering combined with thioflavin S fluorescence to detect fibrils revealed a colocalization of lipids and peptide, but the distribution was not uniform (Fig. 2*A*) (54). High lipid intensities were found in parts of the plaque coincident with high levels of thioflavin S staining. The lipid structures formed were very heterogeneous and included a range of lipid morphologies, including stacked lamellae and larger vesicular structures, covering a large range of sizes up to ∼25 microns. By Raman methods, it was found that the most intense lipid signals colocalized with amide I bands typical of a peptide β-structure, providing strong evidence of peptide–lipid colocalization. Distinctive methylene CH_2_ vibrations characteristic of alkyl chains were found in the absence of vibrations from ester carbonyls or phosphate groups, and without the usual bands seen for cholesterol, consistent with an incorporation of ceramides into the plaque. A transfer of acyl groups to the peptide *via* the lipidation route (35) (Fig. 1) would produce a similar outcome.

Common features of classic cored plaques, revealed by many studies, are that lipids are found in all locations from the core to the periphery of the plaque (53, 54, 59, 60), and there is a lipid-rich annulus around the peptide-rich central core of the plaque (Fig. 2, *B*–*D*). There is evidence that some metals, notably Cu, are enriched in the lipid-rich annulus, whereas others, including Zn and Fe, are enriched in the core (59, 61). The enrichment of lipid in the annulus is relative to both the core of the plaque and the surrounding tissue. It is apparent therefore, that Aβ peptides and lipids colocalize *in vivo* in the center of the plaque, and surrounding this, there is a significant perturbation of the lipid profile relative to the rest of the cell.

### Amyloid fibrils extracted from diseased patients or animal models

Fibrils from a number of sources have been examined after extraction from diseased tissue. In the early days of amyloid research, amyloid preparations were usually associated with significant quantities of lipid. Much of this lipid could easily be removed by chemical extraction, and the lipid was usually viewed as a contaminant to the protein (6, 7). However, in most preparations of Aβ fibrils by this method, some of the lipid proved impossible to remove, leading to residual “contamination” of the fibrils with 1 to 2% by mass of lipid. Indeed, X-ray diffraction studies of some amyloid proteins *ex vivo* also reveal evidence for the presence of lipid (62, 63).

Early studies of the lipid content of amyloid fibrils by TLC, even whilst concluding that lipids were most likely present as a contaminant, noted high contents of cholesterol and fatty acids (6). A mixture of fatty acids was found with a composition that matched the fatty acid profile of the host cell. Neither the high fatty acid content nor the finding that peptides and lipids colocalized in sucrose flotation experiments could be accounted for. Covalent modification to serine residues by a fatty acyl group transferred from the lipid offers one potential explanation for the fatty acid content (35). Such a modification has been observed for other membrane-active peptides (64, 65) and would account for the presence of fatty acids in the fibrils after treatment with ammonium hydroxide should similar modifications to the amyloid peptides be present. Generally, early 20th century studies on amyloid fibrils found lipid contents of 1 to 10% by mass (7). More recently, lipids extracted from a range of sources of amyloid fibril, including AA, ATTR, A*β*_2_M, ALλ, and ALκ amyloidoses (66), a group of conditions resulting from a build-up of amyloid in particular organs and tissues, and AD (paired helical filaments of tau (67)), have been analyzed by high-performance TLC and MS. These preparations typically contain 1 to 16% lipid by dry weight. The major lipids present are cholesterol, sphingomyelin (SM), and sulfatides, plus smaller quantities of cholesterol esters and fatty acids (66). In the case of paired helical filaments, galactocerebrosides and PC lipids are also found. It is notable from both these studies that many components of lipid rafts are associated with fibrils. In the case of paired helical filaments, it is in addition interesting that the fibrils, which are intracellular, are associated with lipids from the outer PM leaflet. This may potentially be a consequence of tau interactions with exosomes, which contain many of the same lipids as rafts (51, 68). Structural studies by cryo-EM of tau filaments extracted from the brains of patients with corticobasal degeneration (69) and AD (70) show the presence of regions weak density around the core β-structure that may reflect the presence of a more dynamic or transiently occupied structure than the rest of the core. These are nonproteinaceous and have been hypothesized to correspond a polyanion in the case of corticobasal degeneration, although they are currently unidentified.

### Aβ fibrils formed in model systems

A number of *in vitro* studies have found evidence for the incorporation of lipids into Aβ fibrils. The addition of Aβ(1–42) protofilaments, formed in the absence of lipids, to supported bilayers of PC/cholesterol/GM1 (68:30:2 by weight) led to significant lipid loss from one leaflet of the membrane in a detergent-like manner (71). Whether the extracted lipids colocalized with the peptide was not investigated by this study. Similar effects were not found for the Aβ(1–42) monomer. A separate study of Aβ(1–42) activity by fluorescence methods on a supported bilayer composed of dioleoyl-*sn*-glycero-3-phosphocholine/SM/cholesterol/GM1 did however note uptake of a general lipid dye into fibrils, as well uptake of the GM1 component. The latter was probed using a GM1-specific marker (72). Lipid uptake was found for addition of either the monomer or the preformed aggregates over the surface.

Aβ(1–40) induces significant perturbation to membranes composed of 1-palmitoyl-2-oleoyl-*sn*-glycero-3-phosphocholine/1-palmitoyl-2-oleoyl-*sn*-glycero-3-phosphoglycerol (POPG)/cholesterol at a P:L of 1:20, leading to changes in the distribution of lipids and ultimately membrane disruption, concomitant with the growth of fibrils (73). At the molecular level, many of the details of this fibrillation, such as fibril morphology and formation kinetics, are significantly affected by whether the monomeric peptide is preincorporated with the lipids prior to membrane formation or added afterward to preformed membranes. For preincorporated samples, short curved fibrils are observed after a period of 4 h, whereas for the peptides added after membrane formation, long and more ordered filament-like fibrils begin to be visible after 46 h. When liposomes containing lipids tagged with a fluorescent label are used, emission intensity changes are observed during fibril formation that are suggestive of an uptake of lipids into the fibrils.

Studies of Aβ(1–40) by solid-state NMR (ssNMR) in a related membrane model containing GM1 in addition to 1-palmitoyl-2-oleoyl-*sn*-glycero-3-phosphocholine/POPG/cholesterol have suggested that the peptide has a detergent-like activity, principally through the observation of isotropic ^31^P signals at 0 ppm, consistent with fast-tumbling aggregates such as micelles (18). However, different results were obtained with total brain lipid extract, which serves to highlight that the outcome of many experiments *in vitro* is highly dependent on the experimental conditions. The presence of isotropic ^31^P peaks in Aβ preparations studied by ssNMR has been noted on other occasions however (73, 74, 75) and may be a common feature in many model systems reflecting a detergent-like activity of the peptide or the formation of lysolipids by lytic processes involving the peptide (35, 76).

### Lipid inclusion into hIAPP fibrils

hIAPP has been well studied in model membranes. The addition of hIAPP to giant unilamellar vesicles (GUVs) labeled with a fluorescent lipid leads to a loss of the tagged lipid from the membrane (77, 78) and the formation of lipid aggregates that may contain peptide (8, 79). The peptide, as with other amyloid forming peptides, has been proposed to exert a detergent-like activity (80). This activity may be inhibited for the monomer in the presence of Ca^2+^ ions but is enhanced for fibrils in the presence of these ions (81).

Characterization of hIAPP fibers formed in a dipalmitoyl-*sn*-glycero-3-phosphoglycerol monolayer by tip-enhanced Raman spectroscopy revealed that the surface of the fibers was highly heterogeneous, although bands were detected that could be attributed to lipids that appeared to be partially coating the surface (82). Coating of hIAPP fibrils by lipids was also observed after administration of the peptide to a supported lipid bilayer (45). Following peptide addition, there was a rapid perturbation of bilayer integrity on a timescale of minutes, followed by the formation of proteinaceous fibrils. Vesiculation was observed at the point of contact between the fibril and the membrane, and fibrils became coated with lipid after a few hours.

**Figure 3:**
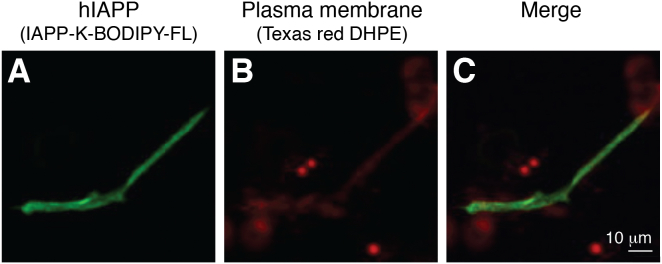
**Colocalization of hIAPP and plasma membrane lipids.** BODIPY-labeled hIAPP (*green channel*) was administered to INS-1E cells in which the plasma membranes were labeled with a Texas red functionalized lipid (*red channel*). Images were acquired after 24 h. *A*, shows the *green* (peptide) channel, (*B*) the *red* (lipid) channel, and (*C*) the merged channels. The scale bar corresponds to 10 μm. Used from Ref. (83) with permission. hIAPP, human islet amyloid polypeptide.

A study of both GUVs and INS-1E cells by confocal microscopy using hIAPP tagged with a fluorescent label found significant colocalization of fibrils with lipids. In GUVs composed of dioleoyl-*sn*-glycero-3-phosphocholine/dipalmitoyl-*sn*-glycero-3-phosphocholine/cholesterol (1:2:1) containing an additional labeled lipid selective for the liquid disordered phase of the membrane, the peptide partitioned into the liquid disordered phase and formed fibrils that incorporated the labeled lipid. In INS-1E cells, using a different lipid label incorporated into the cell membrane prior to administration of the peptide, colocalization was also visualized (Fig. 3) (83).

Overall, there is strong evidence for the incorporation of lipid into fibrils formed by hIAPP.

### α-Syn associates with lipids *in vitro* and *in vivo*

It has long been known that lipids, including SM, are found in LBs, thanks to classic work using lipid-selective stains (84). In fact, we now know that LBs are complex mixtures of materials from a range of subcellular sources (85, 86). Fibrils of α-Syn, when added to neurons and neurites, form LBs over a period of >14 days that incorporate lipids from a range of sources, including organelles (Golgi, endoplasmic reticulum, and mitochondria), the PM, lipid droplets, and lipoproteins. Lipid types include neutral lipids, cholesterol esters, and sphingolipids, amongst others (87). There has been a suggestion that there may not be a direct interaction between α-Syn fibrils and organelles (88), on the basis that fibrils formed by seeded growth in murine neurons, when observed by cryo-electron tomography, do not exhibit the expected abundance of observable membrane contacts. When fibril–membrane interactions were present, they did not produce any apparent membrane deformation. The presence of α-Syn fibrils in LBs, and the relationship between α-Syn fibril formation and LB formation, has also been questioned on the basis of cryo-EM studies of human postmortem brain tissue, in which LBs with immunoreactivity for α-Syn yielded low abundances of fibrils when examined by cryo-EM approaches (86). However, others have cautioned against over-reliance on a single study by cryo-EM methods, partly because of the challenge of representing the whole sample when only a small portion may be analyzed, and partly because although filamentous structures are often observed, their compositions are often not characterized, and so it impossible to conclude whether they are composed of α-Syn (89).

A detailed study of the α-Syn nucleation process examined membrane preparations and soluble cytosolic fractions isolated from rat brain, alongside soluble preparations of human α-Syn obtained by recombinant methods (90). Although the rat peptide was distributed across both soluble and membrane fractions, no free peptide could be detected in the membrane preparations at any time point after isolation, regardless of the salt content of the buffer. This lack of observable dissociation is indicative of a very tight interaction between the peptide and the membrane. Membrane-bound rat α-Syn nucleated on incubation and was able to incorporate soluble human α-Syn added over the top. No aggregation was found in isolated cytosolic fractions, indicating that nucleation occurred in the membrane. In the oligomeric form, but not the soluble monomeric form, both human and rat α-Syn were detected in membrane fractions from sucrose flotation experiments. Coimmunoprecipitation experiments using antibodies specific for rat and human α-Syn produced a similar outcome. Overall, the pattern of behavior was consistent with nucleation occurring in the membrane, leading to the recruitment of oligomers from solution. Costaining of α-Syn aggregates in cells overexpressing α-Syn with an antibody specific for α-Syn and a lipid probe revealed significant colocalization of the two, indicative of significant incorporation of lipid into the α-Syn aggregates.

A key study showed a number of interesting phenomena consistent with lipid incorporation during early α-Syn fibril growth. In anionic membranes containing phosphatidylserines or cardiolipins at pH 5.5, the peptide aggregated in domains after 2 h, with the domain size dependent on the aggregation state. After longer periods, as determined by imaging using a labeled peptide and a labeled lipid incorporated into the GUV membrane, structures growing from the surface of the GUVs were enriched in lipids (91).

Another key study on α-Syn fibrillation in anionic model membranes demonstrated lipid uptake into monomers formed following the addition of the monomer to membranes. Lipid uptake was confirmed by chromatography (TLC) and spectroscopy approaches (NMR). The outcome was dependent on P:L, with close association of lipids to nascent fibers at high P:L, and the absorption of vesicles to formed fibers at low P:L. Lipid uptake into the fibrils was a saturatable process, suggesting the presence of specific interactions between the peptide and lipids, and resulted in morphological changes, such as a conversion from bundles of fibrils to tangles (92).

Other studies have found that α-Syn is able to extract lipids from anionic charged model membranes, including vesicles (93, 94) and supported lipid bilayers (95) leading to visible perturbations to membrane integrity, such as loss of material or the formation of aggregates containing both lipid and β-rich peptide. ssNMR has in addition provided evidence for the coassembly of α-Syn with membranes in vesicles composed of anionic lipids, with the aggregates exhibiting slower but more isotropic rotation of the lipids (96).

### PrP aggregates associate with raft lipids

Monomeric PrP copurifies with fatty acids from hamster brain tissues (97). A number of lipid components have been identified in prion fibers isolated from the brains of rats and hamsters, including SM, galactosylceramides, PC, and cholesterol (98). These are generally assumed to be contaminants carried over during isolation, but nevertheless, the sphingolipids cannot be removed completely by solvent extraction. In a manner that echoes the studies described previously for α-Syn (90) and Aβ (6), a small quantity (about 2%) of PrP with high infectivity is found at low density, in this case, in the meniscus, after sucrose gradient ultracentrifugation of rat brain preparations (98). Despite this high flotation, the levels of lipids in this fraction were very small. Analysis of the lipid content of this fraction by MS revealed a similar profile to the lipids remaining in the fibers after solvent extraction. Nevertheless, the high flotation of the protein could not be accounted for by the low lipid levels. Isolation of PrP^Sc^ from native membranes in styrene maleic acid copolymers has enabled the lipids closely associating with protofilaments to be identified. The lipids identified include cholesterol, PI, and SM. PrP^Sc^ isolated from different animal models exhibits some species-specific profile differences, with, for example, triglycerides present in some but not others (99). Generally, lipids usually associated with lipid rafts are most commonly identified in association with prion aggregates (100).

In a study on model membranes, PrP interacting with GUVs labeled with fluorescent lipids exhibited the formation of extramembranous aggregates containing lipid, although the protein content of these aggregates was not assayed for amyloid character in this particular study (101). Sho, a PrP with similarity to PrP, was observed to form fibrils in model anionic membranes. These fibrils were likely to contain lipids, as evidenced by the decrease in liposome size during their formation (102).

### Other proteins forming lipid-associated aggregates

Additional examples exist where lipids have been found to copurify with fibrils. For example, fibrils of transthyretin from patients with familial amyloidotic polyneuropathy copurify with sphingolipids, phospholipids, acylglycerides, fatty acids, cholesterol, and cholesterol esters. As in other examples described previously, in this particular study, the lipids were treated as contaminants (63). Some proteins, such as the immunoglobulin light chain, show colocalization with lipids and cholesterol *in vivo* but not *ex vivo* or when fibrils are formed *in vitro* (103).

One study identified multiple proteins that formed amyloid in negatively charged membranes *in vitro*, including lysozyme, myoglobin, glyceraldehyde-3-phosphate dehydrogenase, insulin, transthyretin, cytochrome *c*, histone H1, and R-lactalbumin (46). These proteins share the feature of having clusters of cationic residues on their surface. Fiber formation was found to occur very rapidly after mixing (5–10 min), that is, on a timescale much faster than fibrils normally form in many systems *in vitro*. A separate study on cytochrome *c*, however, noted a considerable structural variation in the fibers, both between and within samples, with the protein adopting near-native conformations in some cases (24). Fibers with typical amyloid vibrational spectra were found at high P:L, but fibers with a higher lipid content yielded vibrational bands typical of helical structure and were more heterogeneous. This heterogeneity suggests that rapid modes of forming fibers may not be typical of the process that occur *in vivo* and are different from slower growth processes. It may be the case that the proteins contributing to these rapid forming fibers become trapped in a metastable state if the formation is too fast (24).

A number of studies have identified fibril-like assemblies of proteins with lipids in which the usual properties of amyloid fibrils are not observed. These proteins include some, such as lysozyme, that have previously described as amyloid forming (25, 104, 105, 106), alongside nonamyloidogenic proteins with other forms of membrane activity, such as bovine seminal plasma protein PDC-109 (107) and temporin B, an antimicrobial peptide (49). The seminal peptide PAP(248–286) forms coaggregates with POPG that have some amyloid-like properties, such as binding thioflavin T, but otherwise differ from amyloid forms of the peptide in forming rapidly (<5 min) and being poor at seeding the growth of further amyloid fibrils (108). In addition, these aggregates only form when there is a very high PG content. It has been suggested that the formation of protein–lipid aggregates, including amorphous aggregates, may be general feature of many proteins (49), but the ability of a protein to form aggregates with anionic lipids does not guarantee that these aggregates will be amyloid in nature (105). In some cases, the use of dyes to detect amyloid formation in samples needs to be treated with caution. Thioflavin T, for example, exhibits enhanced fluorescence when bound in hydrophobic environments, such as cell membranes or micelles (109), and Congo red can exhibit nonspecific binding to the membranes of some cells (83).

As a final point, fibrils formed in membranes from apolipoprotein C-II using a labeled short-chain lysolipid (labeled in the headgroup) as a marker led to accelerated fibril formation but no uptake of the label into the fibrils (110). It may well be that there is no lipid uptake in this case. However, this lipid is unusual for this kind of study in being a lysolipid with a short acyl chain and will therefore not partition within the membrane in the same manner as other lipids. The acceleration could be due to transfer of the chain to the protein (35), which would in addition result in loss of the label.

## When and how are lipids incorporated into fibrils?

### The interactions between amyloid fibrils and lipids

It is insightful to examine amyloid structures determined using *ex vivo* material isolated from natural amyloid sources such as plaques and LBs, as many structures determined using samples prepared *in vitro* could not possibly include significant quantities of lipid unless prepared in the presence of an added source of lipid. This argument applies equally to fibrils grown *in vitro* that are seeded using an *ex vivo* amyloid. Fibril morphology is an outcome of competing nucleation processes, which themselves are influenced by numerous other factors, such as PTMs, membrane binding, and the presence of solutes such as anions (23, 27, 111, 112, 113). As a result, most fibril preparations are inhomogeneous, whether formed *in vitro* or isolated from a natural source. Structural variations are often apparent even within a single fibril. Furthermore, a single *ex vivo* seed is able to produce differing morphologies by changing the experimental conditions (114).

As a consequence, fibril structures produced *in vitro* may not reflect the influence of lipids on the morphology during nucleation. Natural amyloids represent structures where the presence of lipids has had the potential to influence morphology throughout fibril formation, even if lipid incorporation into the fibrils is stochastic and substoichiometric. It is a more reasonable assertion that, for a given amyloidogenic protein, a distribution of fibril morphologies characterizes the influence of lipids on fibril formation by that protein. Evidence to support this assertion arises from the observation that polymorphism of fibrils of α-Syn (113, 115, 116), hIAPP (117), Aβ seeded from AD brain amyloid (111, 112) and obtained *ex vivo* (118), plus other proteins (118, 119), is often found within material isolated from the same natural sample. In addition, the morphologies of Aβ filaments and aggregates are influenced by the presence of lipid membranes and their lipid composition (19, 26, 27). The range of morphologies formed by the same protein in the absence of lipids is likely to be different to, but may overlap with, those found in the presence of lipids.

**Figure 4:**
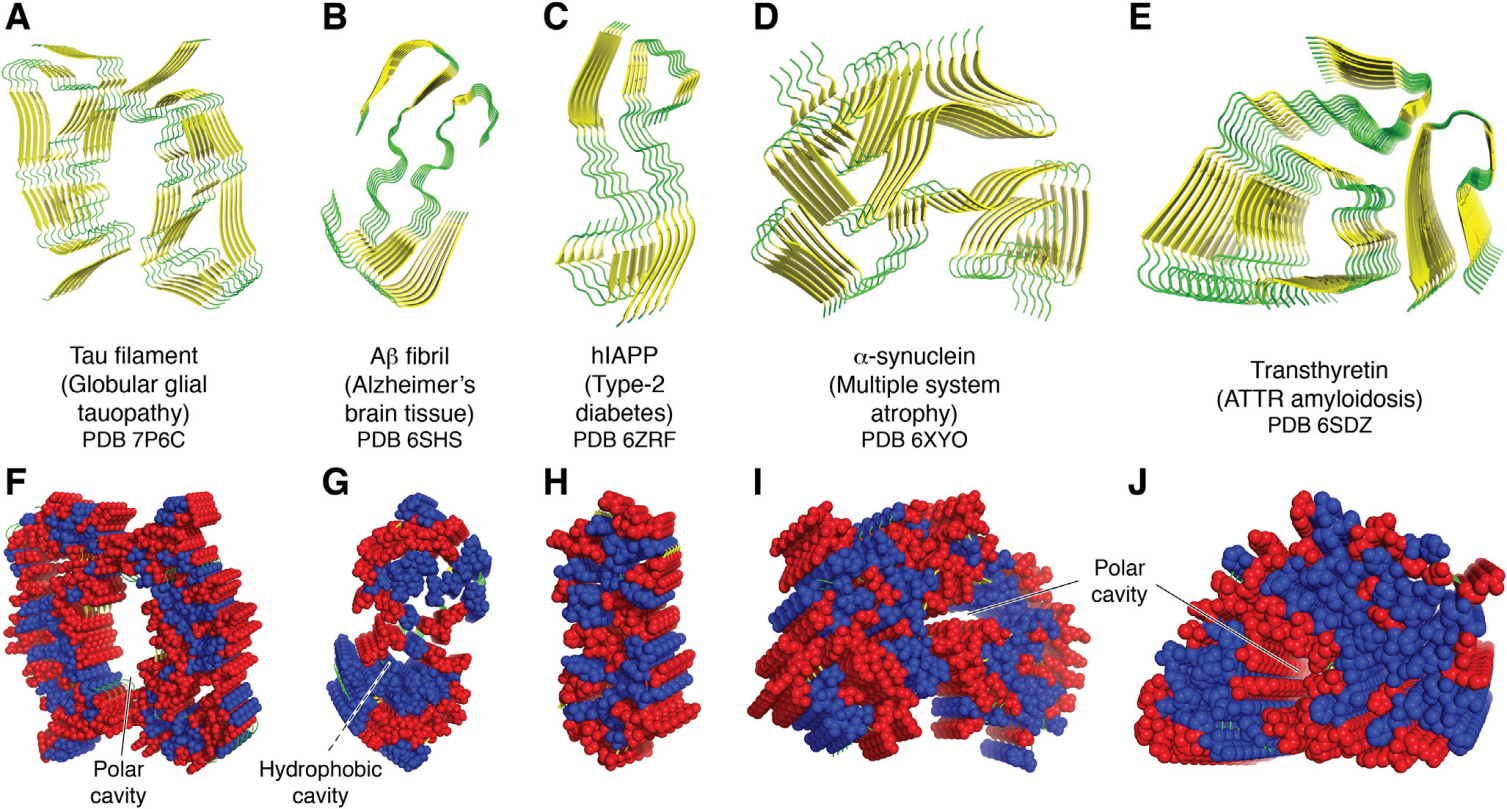
**Illustrative examples of amyloid structures obtained directly from *ex vivo* material by cryo-EM or from amyloid fibrils prepared *in vitro* from an *ex vivo* seed.** In all cases, only one structure from a series of polymorphic forms is presented. *A*, the structure of a tau filament studied by cryo-EM using material extracted from a patient with a globular glial tauopathy (PDB ID: 7P6C (174)). *B*, Aβ isolated from brain tissue of an Alzheimer’s patient (PDB ID: 6SHS (175)). *C*, hIAPP seeded from amyloid extracted from the islet cells of a patient with type 2 diabetes (PDB ID: 6ZRF (117)). *D*, α-synuclein isolated from a patient with multiple system atrophy (PDB ID: 6XYO (115)). *E*, transthyretin isolated from a patient with ATTR amyloidosis (PDB ID: 6SDZ (176)). *F*–*J*, show space-filling representations of the corresponding structures in *A*–*E*. Hydrophobic and hydrophilic residues are colored *blue* and *red*, respectively (with His assigned as hydrophilic and Tyr as hydrophobic). *Solid arrows* indicate the location of polar cavities in the structures. *Dashed arrows* indicate the presence of hydrophobic cavities. hIAPP, human islet amyloid polypeptide; PDB, Protein Data Bank.

In recent years, the structures of several amyloids have been determined *ex vivo*, typically by using cryo-EM on the material itself, or using the material to seed further fibril growth to obtain material for ssNMR studies. All these structures (Fig. 4) reveal the characteristic amyloid motifs, including parallel cross β-sheets and β-helices (120), and typically have a highly folded backbone, often comprising motifs such as S-shaped (16, 23, 114, 121) or Greek key folds (122, 123). Contacts between opposing β-sheets are stabilized by a combination of hydrophobic clustering, including steric zippers, and electrostatic interactions, including salt bridges and Asn-Gln ladders (23, 124, 125, 126, 127, 128, 129). As a consequence, there is a striking segregation of hydrophilic and hydrophobic residues, particularly for structures obtained from fibers prepared *in vitro*. Many of the sites that are available for interaction with lipids are therefore either surface patches, such as those presented at the ends of fibrils, or gaps in the β-sheet structure into which the lipid can closely associate. Structures obtained directly on *ex vivo* material often contain cavities lined with hydrophilic residues. In some structures, these cavities contain polar solutes and anions (113), but in all cases, they are not sites that are able to accommodate the relatively large acyl chains of lipids. It should be remembered however, that lipids are less likely to be resolved in these structures if their inclusion in the fibril is stochastic and therefore not part of a regular matrix. There is also a potential for selection bias in favor of parts of the sample that are more homogeneous and ordered, and therefore likely to give resolvable density, but may contain less lipid.

The observation of fibrils in contact with lipid vesicles in several natural amyloid samples points to a close interaction between the fibril surface and membranes. Examples include Aβ fibrils, which colocalize in plaques with membranes in a diverse array of compositions and forms, ranging from lamellar stacks to vesicles (54), and α-Syn fibrils in LBs, where vesicles include organelles and other lysosome-like structures (87, 85, 86). Electron tomography imaging of material from a cell model for systemic amyloid A amyloidosis reveals a similar heterogeneous mixture of vesicles in contact with fibrils (119). Sites of close interaction between systemic amyloid A fibrils and vesicles are distinguishable by whether they involve contact with the ends of the fibrils, or sites along the fibril length, providing a clear indication that there is more than one mode of interaction between the fibrils and membranes. Interestingly, interaction with the fibril ends is the more common mode, involving around 6% of the fibrils in lipid-rich regions, and leads to deformation of the membrane at the contact site. This deformation is indicative of strong binding interactions at these sites, involving either fibril–lipid interactions, or interactions between fibrils and residual membrane proteins. Molecular level details of the interactions between membrane lipids and intact fibrils are scant. Interactions involving the fibril ends may derive from the initial binding of the amphiphilic peptide monomer with the membrane during nucleation, or be promoted by the presence of PTMs that anchor the peptide to the membrane, such as lipidation (35). Interactions along the fibril length are likely to be weaker if they only involve electrostatic interactions with the lipid headgroups. Some fibrils have hydrophobic patches (23, 130, 131) that could contribute to stronger interactions with the membrane surface if the fibril surface is able to penetrate the membrane interface.

**Figure 5:**
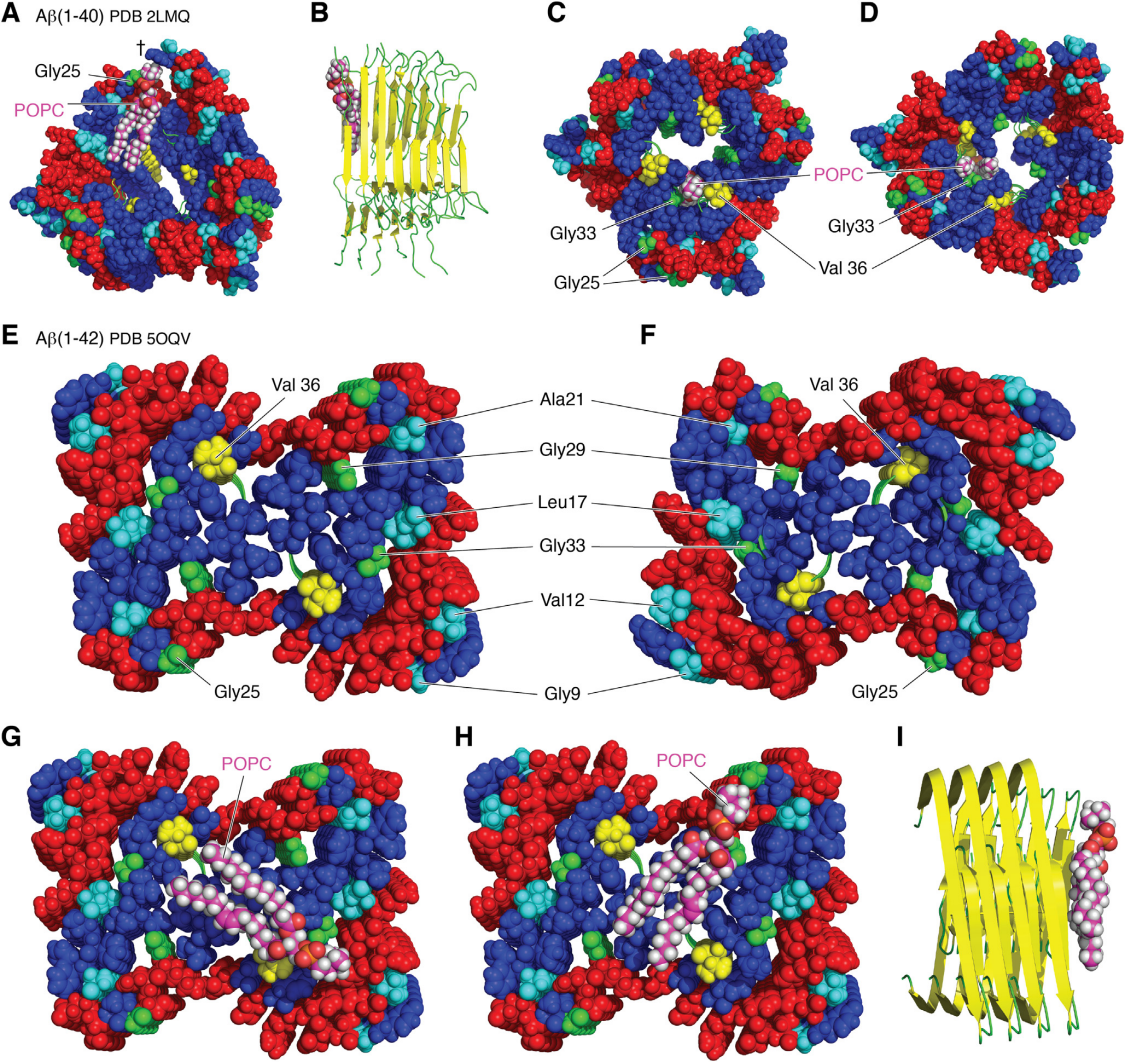
**Representations of potential lipid-binding sites in fibrils of Aβ.** In each case, a molecule of POPC has been manually docked onto a known peptide structure in an orientation and position consistent with proximity data from solid-state NMR measurements in order to illustrate relative size and fit. Hydrophobic and hydrophilic residues are colored *blue* and *red*, respectively (with His assigned as hydrophilic and Tyr as hydrophobic), apart from specific residues that are labeled in each structure. *A* and *B*, show an end view (*space filling*) and side view (*cartoon*) of an Aβ(1–40) structure with spherical symmetry (PDB ID: 2LMQ (135)) with a POPC molecule intercalated in the β-sheet structure. The side chain of Tyr10 is indicated with a *dagger*. *C* and *D*, show the same structure as *A*/*B*, but with a POPC molecule incorporated in the central cavity. Residues 1 to 8 of Aβ(1–40) are missing from this structure. However, residue 9 lies on the outside of the structure away from the central cavity. *E* and *F*, show opposing faces of an Aβ(1–42) fibril composed of two protofilaments (PDB ID: 5OQV (142)) with helical symmetry, looking along the axis of the fibril. *G* and *H*, show POPC molecules docked onto the structure in *E*. *I*, shows a *cartoon* representation of the side view of the structure in *H*. Aβ(1–40), fragment 1 to 40 of the amyloid-β peptide; PDB, Protein Data Bank; POPC, 1-palmitoyl-2-oleoyl-*sn*-glycero-3-phosphocholine.

Sites of potentially much stronger lipid interaction with fibrils have been suggested by a number of ssNMR analyses of amyloid Aβ(1–40)–lipid complexes in aggregates formed when Aβ is preincorporated into liposomes during their preparation. These studies have revealed details of the molecular contacts between the peptide and the lipid (132, 133, 134). Close contacts are found in particular between the phosphorous atom of the lipid and the backbone carbonyl groups of residues Gly25 and Val36, with marginally weaker contacts to Gly29 and Gly33. No significant contacts are observed between the lipid headgroup and Gly9, Val12, Leu17, and Ala21. In general, the close interactions involve the loop segments of the core β-structure and the lipid headgroups, suggesting that lipids intercalate into the hydrophobic core of the sheets, disrupting the hydrogen bonding network (Fig. 5).

Similar to fibrils isolated from natural amyloid deposits, fibrils prepared *in vitro* from purified proteins or peptides are well known to exhibit a range of polymorphic forms that are adopted according to their conditions of preparation (17, 20, 26, 114). Fibrils formed from pairs of filaments typically have either rotational (*C*_2_) or screw (2_1_) symmetry with respect to the fibril axis. In some Aβ fibrils with rotational symmetry, such as those with threefold symmetry (Fig. 5, *A* and *B* (135)), the outer sheet overhangs the inner sheet by two β-strands in the repeating unit along the fibril axis, providing a potential lipid interaction site. In line with the intercalation described previously, the head group of a lipid molecule is able to interact with Gly25 and the hydrophobic cluster in the center of the structure whilst occupying a position that has a good complementary fit with the inner strand structure. This interaction also facilitates other favorable noncovalent interactions, such as a cation–π interaction between the quaternary ammonium group of the lipid and the side chain of Tyr10. The rotational symmetry leads to equivalent interaction sites at each end of the repeating unit, leading to multiple potential interaction sites along the length of the fibril. Incorporation of lipids into filaments by intercalation in this manner would potentially yield structures with noninteger numbers of molecules per repeat, a scenario that has some precedent in filaments of Aβ and yeast PrPs, albeit for filaments formed in the absence of lipids (136).

This fibril morphology with a threefold rotational symmetry is also of interest because it appears to have a hydrophobic cavity large enough to accommodate one or more lipids whilst permitting close contact between the lipid headgroup and key residues (Fig. 5, *C* and *D*). It may be that fibril growth in the presence of lipids influences growth pathways in favor of morphologies with larger hydrophobic cavities to accommodate lipids. It is notable that this threefold symmetry has been found for fibrils seeded from patients with AD (137). A hydrophobic cavity large enough to accommodate lipids in a predominantly β-sheet structure has a precedent in PM–endoplasmic reticulum lipid transport proteins (138, 139, 140, 141). If lipid movement was permissible along the hydrophobic cavity of an amyloid filament, it would provide a means for lipid incorporation into growing filament ends that are distal from the membrane surface.

For fibrils with helical symmetry, the fibril ends are nonequivalent (142). Interestingly, this nonequivalence extends to the residues involved in the close contacts with lipids described previously (Fig. 5, *E* and *F*). On one face (Fig. 5*E*), the interacting residues are accessible, whereas on the other face (Fig. 5*F*), they are partially buried. Furthermore, these residues on the accessible face are positioned in a manner close to the hydrophobic cluster in the core of the fibril that would permit simultaneous contact of the headgroup with the known contact sites determined by NMR and contact of the lipid acyl chains with the hydrophobic fibril core (Fig. 5,*G*–*I*). Should steric restrictions favor lipid interaction at one fiber end, lipid binding would have the potential to restrict fibril growth from that axis, thereby directing fibril growth from the other axis, with downstream effects on morphology and other structural features such as branching and fragmentation (Fig. 1). The differential incorporation of lipids into fibril ends to influence the growth axis may produce the variations in the fibril network observed in some samples, exemplified by serum amyloid A1 fibrils obtained in a cell model (119).

### Lipid incorporation into fibrils

In the early stages of formation, interactions with membranes can promote nucleation, but there is limited opportunity for uptake of lipid material at this stage. The only available routes for uptake of parts of lipids are either by transfer of a fatty acyl group to a serine residue or a lysine residue of the peptide (35) or by transfer of a reactive aldehyde fragment derived from oxidative damage to a lipid (33). In some cases, the monomeric peptides or amyloid seeds may be able to exert a detergent-like activity, leading to loss of membrane material or remodeling of the membrane. Protein–lipid mixtures at this stage are a dispersion of protein in a predominantly lipid matrix. This stage of the process *in vivo* corresponds to the formation of diffuse plaques in AD (Fig. 1*A*).

Lipids are most likely to begin to associate with the nascent β-structures in the intermediate stages of fibril formation, during which oligomers and protofibrils begin to form (8, 78, 79, 91, 92). A recent study of Aβ fibril formation in flow conditions concluded that early forming fibrils and membranes form an equilibrium between the membrane-bound peptide and peptide–lipid aggregates in solution. This process implicitly favors the incorporation of lipids into fibrils at an early stage of fibril formation (143). This stage of the process is where the most toxic membrane-active species form and corresponds to the formation of compact structures in AD (Fig. 1*B*). Lipids incorporated into fibrils at this stage are likely to be tightly associated within the structure and correspond to those that remain following solvent extraction protocols on amyloid deposits isolated from diseased tissues. These lipids correspond to those incorporated into the structure by intercalation or inclusion in a hydrophobic cavity.

Larger fibrils, formed in the later stages of amyloid assembly, are generally less toxic than the early forming low–molecular weight assemblies. When preformed, these fibrils often exhibit low affinity for lipid membranes and yield little or no evidence of lipid uptake when added to membranes (66, 78). Those that do interact with membranes bind in a peripheral manner and either attach as inert structures (133, 134) or become coated in lipids adsorbed from the membrane (45, 72, 77, 92). These lipids are likely to be loosely associated and easily removed by chemical extraction of material isolated from amyloid deposits. This stage of fibril formation corresponds to the classical core structure in AD (Fig. 1*C*), and the lipids are likely to be those in the lipid annulus around the protein core (Fig. 2).

It is finally worth remarking on the effects of lipids on the mechanical properties of fibrils. A systematic comparison of the effects of fibril formation in the presence and absence of lipids provides evidence that lipids can have a direct effect on the mechanical strength of fibrils. Fibrils formed by a fragment of serum amyloid A1 that were either isolated from their natural source or prepared *in vitro* from *ex vivo* seeds had a similar bending rigidity, as assessed by the persistence length of the fibrils. Amyloid fibrils formed by the same protein *in vitro* without seeding, or without the presence of lipids, formed fibrils with a higher bending rigidity (119). These observations suggest that lipid inclusion in the early stages of nucleation favors morphologies that are “softer” than protein-only fibrils formed *in vitro*, potentially as a consequence of having a higher helical content (144). A consequence of this lower mechanical strength for fibrils formed in the presence of lipids would be an increase in the rate of fibril fragmentation, with a concomitant increase in the rate of fibril growth (145).

## Concluding remarks

It is apparent that, far from being the contaminants described in early preparations of amyloid fibrils, lipids are an integral part of most fibrillar deposits that form *in vivo* in association with disease states. Considering the evidence for the presence of lipids in amyloid fibrils across several amyloid-forming peptides, some common themes emerge. Many of these peptides associate with membranes as amphipathic helices and form fibrils in anionic membranes, usually with similar kinetics of nucleation and growth (hours to days). When studied *in vivo*, fibrils can be localized to structures rich in lipids and are isolated alongside lipids when extracted from diseased tissues, typically at a level of 1 to 15% lipid by dry mass. Chemical extraction of deposits from multiple sources fails to remove all traces of lipid, typically leaving behind 1 to 2% by mass. Many of these peptides, when separated by sucrose flotation, equilibrate to lower densities than expected. In model systems, these peptides can be demonstrated to form coaggregates with lipids. In favorable circumstances, there is clear evidence that the protein is in fibrillar form and that lipids are present. It seems likely that the presence of lipids in amyloid deposits is fundamental to the biology of these structures, particularly as membranes are known to influence the morphology and material properties of amyloid fibrils. The role of lipids in these structures is therefore worthy of further study.

## Conflict of interest

The author declares no conflicts of interest with the contents of this article.
